# A labeled synthetic mobile money transaction dataset

**DOI:** 10.1016/j.dib.2025.111534

**Published:** 2025-04-07

**Authors:** Denish Azamuke, Marriette Katarahweire, Engineer Bainomugisha

**Affiliations:** Department of Computer Science, Makerere University, Plot 56 Pool Road, P. O. Box 7062, Kampala, Uganda

**Keywords:** Financial data, Digital wallet, Phone-based payments, Simulated records, Fraud detection

## Abstract

This data article introduces a labeled synthetic mobile money transaction dataset created using MoMTSim, a multi-agent-based simulation platform designed and validated specifically for mobile money transactions. MoMTSim toolkit simulates mobile money interactions, ensuring that the generated synthetic dataset closely mimics the statistical properties of real transaction data. This dataset encapsulates a wide range of transaction features, such as timestamps (step), transaction amounts, the initial and new account balances of both the initiator and recipient, participant IDs, and the types of transactions conducted. The included transaction types span deposits, withdrawals, transfers, payments, and debits. Each record in the dataset also carries a label that identifies whether the transaction is legitimate or fraudulent. The synthesis of this dataset using MoMTSim is described in this article and its structure and summary statistics are also presented. The dataset is particularly suitable for training and testing machine learning algorithms to detect financial fraud. Additionally, it holds the potential for benchmarking fraud detection algorithms and systems and validating synthetic data generation methodologies.

Specifications Table

**Table:** 

Subject	Data Science
Specific subject area	Applied Machine Learning
Type of data	CSV files of raw synthetic mobile money transaction data.
Data collection	The dataset was generated using the MoMTSim toolkit, which simulates mobile money transactions involving clients, banks, merchants, and fraudsters. It incorporates common fraud typologies such as account takeover fraud in the form of SIM swaps and lost credentials, refund fraud, and PII (email, phone, and national identification number) sharing. MoMTSim models various transaction types such as deposits, withdrawals, and payments based on rules and probabilities derived from analyzing a large real transaction dataset, producing synthetic transaction logs (labeled transaction datasets).
Data source location	Institution: Department of Computer Science, Makerere University. City/Town/Region: Kampala/Eastern Africa region. Country: Uganda.
Data accessibility	Repository name: Mendeley Data Data identification number: DOI: 10.17632/zhj366m53p.2 Direct URL to data: https://data.mendeley.com/datasets/zhj366m53p/2
Related research article	D. Azamuke, M. Katarahweire, E. Bainomugisha, MoMTSim: A Multi-Agent-Based Simulation Platform Calibrated for Mobile Money Transactions, IEEE Access, vol. 12, pp. 120226-120238, 2024. 10.1109/ACCESS.2024.3439012

## 1 Value of the Data

• This dataset was generated to mirror real-world mobile money transaction behaviors since the simulator was calibrated using real data. The dataset simulates different types of fraudulent transactions, including scenarios such as account takeover fraud, refund fraud, and fake credentials. This breadth of fraud scenarios is uncommon in several existing datasets. By comparing distribution shapes, and transaction patterns with real transaction data (as discussed in the original MoMTSim paper [1]), we ensured that the synthetic data exhibits realism in transaction characteristics, and this makes the dataset a valuable resource, and it can be used for training and testing fraud detection methods.
• Real mobile money transaction data can be proprietary due to financial regulations and user privacy requirements. This labeled synthetic transaction dataset fills this gap by offering an openly available resource that researchers and practitioners can use to prototype, train, and benchmark fraud detection algorithms in a manner not feasible with confidential real-world data.
• Researchers from finance, computer science, and data analytics can use this dataset for interdisciplinary research including investigating fraud detection mechanisms, behavioral finance models, and predictive analytics techniques. Since it is synthetic yet representative, the dataset can provide a “sandbox” for testing ideas that could later be adapted to real-world settings.
• Beyond fraud detection use cases, this dataset showcases a methodology for generating synthetic financial transaction data that resembles real data. Researchers can adapt and extend these data generation techniques to other transactional or tabular data scenarios.

## 2 Background

The synthetic mobile money transaction dataset was compiled to support the testing and evaluation of financial crime detection algorithms within the mobile money ecosystem, particularly in East Africa, where mobile money is critical for financial inclusion. Due to the high transaction volumes and significant financial inclusion rates, mobile money services are vital for everyday commerce and savings in this region. However, the prevalence and significance of mobile money also amplify the risks and potential for financial fraud, paving the way for research in fraud detection [1,3,4]. The dataset was generated using MoMTSim^1^, a multi-agent-based simulation platform calibrated to mirror real-world financial transactions in the realm of mobile money service. This platform allows for dynamic adjustment of parameters to produce diverse, realistic transaction datasets, which include typical user behaviors as well as anomalous activities indicative of financial fraud. This dataset [5] directly complements and extends the research presented in the original MoMTSim [1] study by providing a labeled synthetic dataset enriched with diverse fraud scenarios that can be used for training machine learning algorithms. Additionally, researchers and practitioners in this domain can utilize this dataset as a ready-to-use resource to evaluate new financial crime control methods and systems and improve synthetic data generation methodologies.

## 3 Data Description

The data consists of labeled synthetic transactions generated using the MoMTSim [1] toolkit. MoMTSim models the mobile money ecosystem consisting of clients, banks, merchants (supermarkets, fuel stations, and other businesses that accept mobile money payments), and mobile money agents (also called mobile money merchants). The transaction types in the simulated dataset include deposit, which is concerned with a user loading electronic money on their mobile money account via a mobile money merchant or agent. Withdrawal is the opposite of deposit (removes money off mobile money account), transfer involves a user sending money from their account to another account within the mobile money network. Payment is concerned with the purchase of goods or services, while debit involves a user moving electronic money from their mobile money account to a bank account. The various transaction types in the simulated data are summarised in Table 1.

**Table 1:** Transaction types in the simulated mobile money transaction data.

Transaction type	Description
Deposit	Loads electronic money into a mobile money account
Withdrawal	Removes electronic money from a mobile money account
Payment	Facilitates purchase of a good or service using mobile money (a user buys a good or service using their electronic funds)
Transfer	Enables the exchange of electronic funds among users within the mobile money platform (a user sends electronic funds to another user)
Debit	Moves electronic money from a mobile money account to a bank account

**Fig 1:**
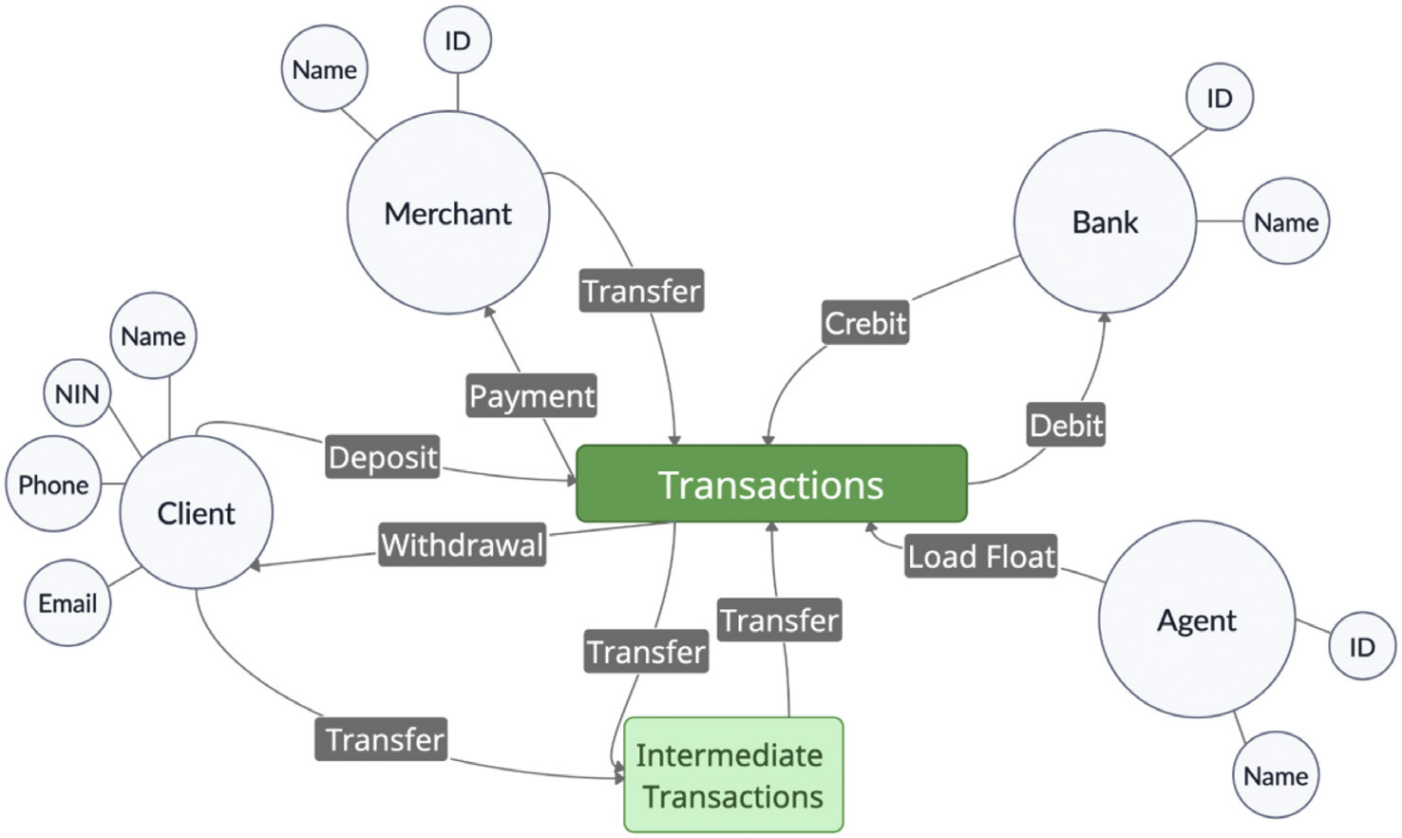
Mobile money financial ecosystem.

Another transaction type in the real mobile money financial ecosystem includes credit, which is concerned with the movement of electronic money from a bank account to a mobile money account. This type of transaction is uncommon due to its high charges in the real financial ecosystem. Users often prefer transactions that are carried out within the mobile money platform. Transactions on the mobile money platform due to interactions of the actors form a social network and this financial ecosystem is shown in Fig. 1.

Mobile money merchants (agents) play a passive role in facilitating deposit and withdrawal transactions by loading electronic funds onto the platform as float. The float is used to support clients and other actors in the conversion of cash into electronic money and vice-versa. Clients can deposit, withdraw funds, pay for goods and services, or transfer funds to other clients. Merchants can receive payments, and carry out transfers on the platform while banks can receive funds from mobile money clients. Unlike related studies on the simulation of multi-agent financial systems including PaySim [4], MoMTSim [1] introduces an iterative algorithm used for calibrating several parameters of the simulation model. The iterative algorithm allows identification of missing behaviors and introduction of new behaviors, removal of errors, and preservation of agent behaviors that match the behaviors in the real ecosystem. This improves realism in simulations and accuracy of the resulting synthetic datasets. Besides, MoMTSim models diverse fraud scenarios including account takeover fraud in the form of SIM swaps and lost credentials, refund fraud and use of fake credentials to create mobile money financial accounts. This depicts the diversity of fraudulent activity in the real financial ecosystem, improving on the modeling of single fraud scenarios by related works [4].

This data article presents two versions of the synthetic mobile money transaction dataset. Dataset version 1 has 1,720,181 labeled transaction records that can be used for relatively small experiments such as training and testing classical machine learning algorithms for fraud detection. The percentage total amount of transactions per transaction type in dataset version 1 includes 58.94 % deposits, 13.49 % withdrawals, 26.33 % transfers, 1.20 % payments, and 0.04 % debits. The second dataset, dataset version 2 contains 4,225,958 labeled transaction records and it can be used for training and testing state-of-the-art fraud detection models. Dataset version 2 has 48.21 % deposits, 9.91 % withdrawals, 38.23 % transfers, 3.32 % payments and 0.33 % debits. The proportions of each transaction type simulated in the datasets are presented in Table 2. Table 2 shows the transaction counts and total transaction amounts per transaction type in the datasets.

**Table 2:** Proportions of transaction types in the simulated mobile money data.

Dataset Version 1 (1,720,181 transaction records)			
Transaction type	Count	Total amount	% Total amount
Deposit	384,431	53,264,660,000	58.94
Withdrawal	93,785	12,196,070,000	13.49
Transfer	569,328	23,796,880,000	26.33
Payment	667,245	1,081,122,000	1.20
Debit	5,392	37,308,750	0.04
Dataset Version 2 (4,225,958 transaction records)			
Deposit	8,824	108,020,500,000	48.21
Withdrawal	27,064	22,202,420,000	9.91
Transfer	2,510,947	85,659,650,000	38.23
Payment	1,677,798	7,449,373,000	3.32
Debit	1,325	734,252,000	0.33

The features in the two versions of the data are the same and they include the transaction amount, the initial and final balances for the initiator and recipient of a transaction. The transaction type, timestamp (step) with a step mapping to an hour in the real world, IDs for the initiator and recipient of a transaction, and a transaction label (1 for fraud and 0 for legitimate transaction). These features of the data are summarised in Table 3.

**Table 3:** Structure of the synthetic mobile money transaction data.

Feature	Description
step	Unit of time in the simulation environment, 1 step is equivalent to 1 hour in the real world
transactionType	The type of transaction: deposit, withdrawal, transfer, payment, or debit
amount	Amount of transaction
initiator	ID for the initiator of a transaction
oldBalInitiator	Initial account balance for the initiator
newBalInitiator	New account balance for the initiator
recipient	ID for the recipient of a transaction
oldBalRecipient	Initial account balance for the recipient
newBalRecipient	New account balance for the recipient
isFraud	Label 1 for fraud and 0 for legitimate transaction

## 4 Experimental Design, Materials and Methods

The MoMTSim [1] toolkit was used to generate synthetic mobile money transaction datasets. The toolkit simulates the mobile money financial ecosystem presented in Fig. 1 comprising various types of agents, including clients, merchants, banks, and fraudsters. The simulation was configured to run for 720 time steps, where each step represents one hour, approximating one month of real-world activity. The parameters used to generate these datasets were derived from a large real transaction dataset to ensure realistic behavior profiles.

The simulation model of MoMTSim was designed using MASON [2], a generic multi-agent simulation toolkit in Java, capable of supporting large-scale custom simulations. Key parameters of the simulation model included the number of each agent type (clients, banks, merchants), probability distributions for controlling fraudulent and legitimate agent behaviors, transaction types, and financial constraints (transfer transaction limits, overdraft thresholds). Diverse fraudulent activities such as account takeover fraud, refund fraud, and the use of fake credentials were incorporated into the simulation model, each controlled by parameters that determine the likelihood and dynamics of these behaviors. These fraudulent behaviors are described as follows:

• **Account takeover fraud.** A common fraud typology within the mobile money financial ecosystem is account takeover fraud in the form of SIM swaps and lost credentials. In a SIM swap fraud, a criminal replaces a customer's SIM card, gaining control over the phone number linked to the customer's mobile money account. This unauthorized access allows the fraudster to conduct transactions and potentially drain the victim's account through multiple withdrawals executed via mobile money merchants. Lost credentials occur when a customer's phone is lost, enabling fraudsters to access and potentially change the short PIN code that secures the mobile money account, thus taking control of the victim's gadget that can ultimately be used to carry unauthorized, numerous mobile money transfers.
• **Refund fraud.** In refund fraud, a fraudster makes payments for goods or services to a retailer and repeatedly requests refunds. They target vulnerable merchants and engage in multiple transactions, potentially involving new victims. The fraudster eventually withdraws the funds directly from their account or through intermediaries known as mules.
• **Fake credentials.** In this typology, a fraudster establishes a mobile money account using someone else's phone number, email, or national identification number (NIN). The account is then used by the fraudster to carry out fraudulent transactions, falsely representing the individual linked to the identification details provided.

Every simulation in MoMTSim used the following procedure to output synthetic mobile money transaction datasets.

• **Initialization of input parameters and data.** The simulation loads configuration files containing parameters for the number of clients, merchants, banks, and fraudsters, as well as the total number of simulation steps and a random seed for reproducibility. At this stage, input CSV files providing transaction types, initial balance distributions, client profiles, transaction occurrence limits, and overdraft constraints are also loaded. A directory containing definitions and parameters for various fraud typologies is also specified, among other things.
• **Agent initialization**. Based on the input parameters, the simulation instantiates a defined population of agents including the clients, merchants, banks, and fraudsters. Each agent is assigned initial conditions (balance distributions for clients) and behavioral rules derived from the statistical properties of large real transaction datasets.
• **Simulation execution.** The simulation runs for a predefined number of steps (720). At each step, agents interact to produce transactions (deposits, withdrawals, transfers, payments or debits), with participants selected probabilistically depending on the loaded statistical distributions. Also, fraudsters may engage in fraudulent behaviors, including account takeovers, refund fraud, or using fake credentials, according to the assigned parameters for fraud likelihood and target selection. Besides, transaction constraints such as maximum transfer amounts and financial rules (how deposits and withdrawals affect account balances for initiator and recipient) are enforced throughout every simulation.
• **Data recording.** Every generated transaction, whether legitimate or fraudulent, is recorded in a CSV file forming the synthetic transaction data. This recording of the data includes transaction details (transaction type, initiator, recipient, amount, timestamp, and fraud status for every transaction) thus capturing a complete record of the simulated financial activity.

For reproducibility, simulations were configured with a fixed random seed. Statistical validation methods, including the sum of squared errors, the Kolmogorov-Smirnov test, and Bland-Altman plots, were used to confirm the realism and validity of the generated synthetic mobile money transaction data as presented in the original MoMTSim study [1]. Post-simulation analysis was performed using Python's pandas library to load and process the CSV output into DataFrames.

## 5 Materials and Methods

The MoMTSim toolkit, built on the MASON multi-agent simulation framework [2], was used to model the interactions among simulated agents including banks, clients, merchants, and fraudsters. The simulation was executed on a MacBook Pro equipped with an M1 Pro chip processor and 16 GB of RAM. Two versions of synthetic mobile money transactions were generated using varying populations of agents in MoMTSim as shown in Table 4. Table 4 provides the simulation configurations for two simulations. These configurations include the number of steps, which is the total unit of time, a multiplier for rescaling numerical values, and a seed to manage the reproducibility and randomness of simulations. Parameters for agents such as the number of clients, banks, and merchants in order to have a definite population in a simulation. All simulations used CSV files containing initial balance distributions for clients, client profiles, and aggregated mobile money transactions. Other loaded files included CSV files specifying transaction types, maximum occurrences per client, and overdraft limits. A folder providing the descriptions and parameters for each fraud scenario being simulated. Agents interacted in MoMTSim based on defined probabilities and parameters, generating synthetic transactions such as deposits, withdrawals, transfers, debits and payments.

**Table 4:** Simulation configurations used in generation of synthetic mobile money transaction data.

Simulation Parameter	Dataset Version 1 (1,720,181 records)	Dataset Version 2 (4,225,958 records)
nbSteps	720	720
seed	1,000	1,000
multiplier	1	1
nbClients	3,000	6,000
nbFraudsters	1,000	2,000
nbBanks	10	20
nbMerchants	1,500	3,000
transferLimit	20,000,000,000	20,0000,0000,000

**Fig. 2:**
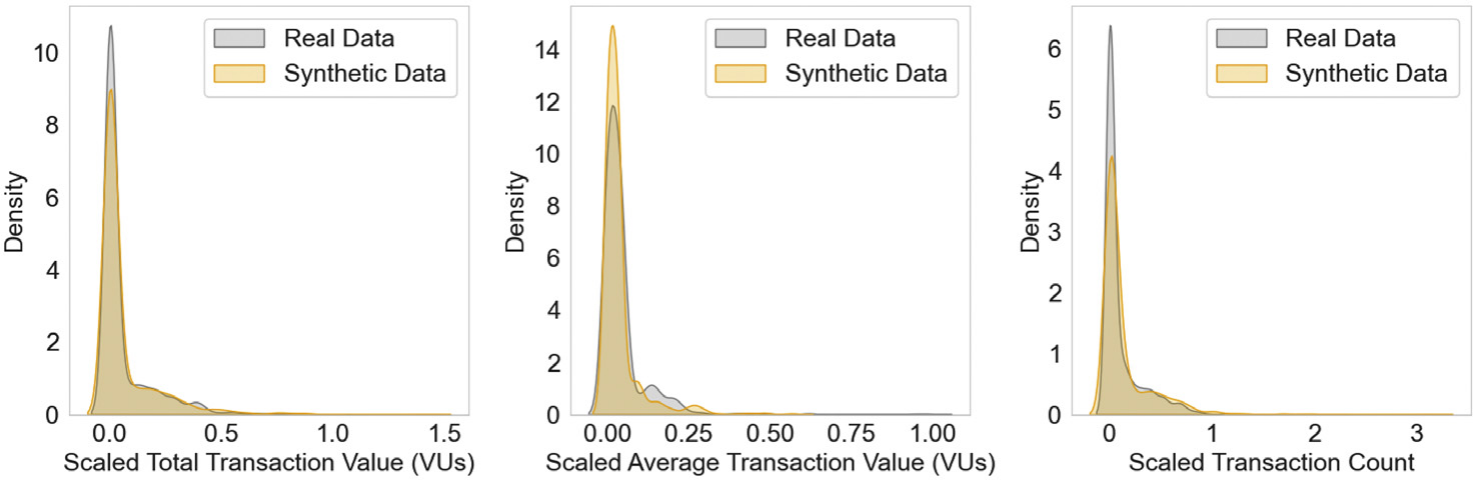
Resemblance of synthetic data to real data [1].

CSV output files of synthetic transactions containing the required fields (timestamps, agent IDs, transaction amounts, transaction types, and fraud indicators) form the labeled data described in this study, though other log files including parameter file history, synthetic client profiles, and synthetic aggregated transactions were also generated. In the original MoMTSim paper [1], we demonstrated that our synthetic datasets closely match the statistical properties of real data. Fig. 2 highlights this similarity by showing substantial overlap between the real and synthetic distributions of total transaction value, average transaction value measured in virtual units (VUs), and transaction count. Although minor discrepancies appear in the peak and tail densities, overall distribution shapes remain highly similar. Further details on these comparisons and supporting metrics can be found in the original MoMTSim paper [1].

The output dataset with 1,720,181 records (version 1) has 89.80 % legitimate transactions and 10.20 % fraudulent transactions. The dataset with 4,225,958 records (version 2) has 47.16 % legitimate transactions and 52.84 % fraudulent transactions. The proportions of each transaction class (fraud and legitimate) in the datasets are summarised in Table 5. The datasets provide different class distributions that may be useful for various research purposes, and these differences arise due to the varying simulation configurations as shown in Table 3. This dataset [5] extends MoMTSim [1] for fraud modeling.

**Table 5:** Distributions of synthetic mobile money transactions in the simulated data.

Class of transaction	Count	% Count	Count	% Count
Dataset	Version 1 (1,720,181 records)		Version 2 (4,225,958 records)	
Legitimate	1,544,663	89.80	1,992,840	47.16
Fraud	175,518	10.20	2,233,118	52.84

## Limitations

The labeling of transactions as fraudulent or legitimate was based on predefined rules and scenarios, which may not encompass all possible types of fraud. This limits the effectiveness of this dataset in training models for detecting evolving fraud tactics that were not anticipated during the simulation design. Besides, this dataset has not been evaluated for its utility in training or testing machine learning models since this is out of scope of this paper. Future studies could explore how models trained on this dataset perform on real-world test sets.

Mobile money ecosystems are rapidly evolving, potentially introducing significant new transaction types. However, this dataset is confined to five transaction types which include deposits, withdrawals, transfers, payments, and debits.

## Ethics Statement

The authors have adhered to ethical guidelines and publication requirements for Data in Brief. This dataset did not involve human subjects, animal experiments, or data from social media platforms.

## CRediT Author Statement

**Denish Azamuke:** Methodology, Data Curation, Validation, Writing - Original Draft, Writing - Review & Editing. **Marriette Katarahweire:** Project administration, Writing - Review & Editing, Supervision. **Engineer Bainomugisha:** Conceptualization, Funding acquisition, Writing - Review & Editing, Supervision.

## Data Availability

Mendeley DataSynthetic Mobile Money Transaction Dataset (Original data).

## Footnotes

1 MoMTSim toolkit: https://github.com/aiinfinancegroup/MoMTSim.
